# Socioeconomic Gauging of Brown and Levy Power Motions

**DOI:** 10.3390/e28020216

**Published:** 2026-02-12

**Authors:** Iddo Eliazar

**Affiliations:** School of Chemistry, Tel Aviv University, Tel Aviv 6997801, Israel; eliazar@tauex.tau.ac.il

**Keywords:** selfsimilar processes, sub-diffusion and super-diffusion, aging and anti-aging, persistence and anti-persistence, fractal wealth distributions, Lorenz curves, Gini index, 02.50.-r (probability theory, stochastic processes, and statistics), 05.40.-a (fluctuation phenomena, random processes, noise, and Brownian motion), 89.65.-s (social and economic systems)

## Abstract

Recently introduced, power Brownian motion and power Levy motion are versatile and practical anomalous-diffusion models. On the one hand, the power motions are easily constructed and are easily tracked. On the other hand, the power motions display an assortment of anomalous behaviors including: sub-diffusion and super-diffusion; aging and anti-aging; and persistence and anti-persistence. This paper investigates the power motions from a socioeconomic-inequality perspective. Using this perspective, key statistical and temporal behaviors of the power motions are interpreted and scored. In particular, the paper provides simple and explicit quantitative answers–which are based on socioeconomic inequality indices–to the following question: what is the ‘degree of anomaly’ of each of the power-motions’ anomalous behaviors? The socioeconomic approach presented in this paper may be applied (in future research) to additional anomalous-diffusion models.

## 1. Introduction

Fractals are objects that are invariant with respect to ‘zooming in’ and ‘zooming out’ [1,2,3]. Fractality appears naturally in the context of physical diffusion processes [4,5,6,7]. Indeed, such processes ‘run’ on the molecular level, and they are observed on the human level. In the transition from the molecular level to the human level, fractality emerges universally [8].

Following the works of Ingenhousz [9], Brown [10], and Bachelier [11,12], the theory of physical diffusion processes began with the works of Einstein [13] and Smoluchowski [14]. The theory’s keystone is the foundational Brownian-motion model [15,16,17]. Further works [18,19,20,21,22] unveiled the fact that beyond the dominion of ‘regular’ diffusion processes there is a vast realm of ‘anomalous’ diffusion processes [23,24,25,26,27,28,29,30,31,32,33,34,35].

Since Einstein and Smoluchowski, via the discovery that diffusion comes in both regular and anomalous ‘flavors’, the interest in diffusion processes is extensive and ongoing. Recent examples of anomalous-diffusion studies include: higher-order diffusion [36]; origins of diffusion [37]; robust criterion of diffusion [38]; convolutional transformers [39]; self-propelled particles [40]; change-point detection [41]; power-law-correlated noises [42]; elastic gels [43]; super paramagnetic walkers [44]; free-ranging birds [45]; conical channels [46]; fractal homogenization [47]; and shear-driven anomalous diffusion [48].

(The wide span of topics covered by the above examples demonstrates the highly multi-disciplinary nature of diffusion research. Many more examples of anomalous-diffusion works will be noted along this paper.)

Among the recent studies on diffusion theory are the models of *power Brownian motion* (PBM) [49,50,51] and *power Levy motion* (PLM) [52,53,54]. The word “power” in the names of these models is due to the following fact: the ‘pillars’ of PBM and PLM are certain power-laws, and these power-laws define PBM and PLM.

PBM is the unique fractal diffusion model that is Gaussian and Markovian [50,55]. The white noise that ‘drives’ PBM is Gaussian, and its statistical fluctuations are ‘mild’. Elevating from Gaussian white noise to Levy white noise–whose statistical fluctuations are ‘wild’–yields PLM. As PBM, also PLM is a fractal and Markovian diffusion model.

On the one hand, PBM and PLM are easily constructed and are easily tracked. On the other hand, PBM and PLM display a host of statistical behaviors including: sub-diffusion and super-diffusion; aging and anti-aging; and persistence and anti-persistence. Combining together analytic simplicity and statistical riches, PBM and PLM have a significant potential for practical applications. As these models were introduced only recently, applications are yet to be proposed.

The purpose of this paper is to illuminate PBM and PLM from a rather unexpected perspective: *socioeconomic inequality* [56,57,58,59,60]. To that end let’s shift from fractality in the context of physical diffusion to fractality in the context of wealth distributions (in human societies).

Fractal wealth distributions [61,62] are defined by a certain power-law with a positive power *p*. Depending on the value of the power *p*, the fractal wealth distributions are classified as follows.

When the power is smaller than one (p<1) then the fractal invariance is with respect to ‘zooming in’ on the rich [61,62], and hence: the wealth distribution among the rich is statistically identical to the wealth distribution among all society members. When the power is larger than one (p>1) then the fractal invariance is with respect to ‘zooming in’ on the poor [61,62], and hence: the wealth distribution among the poor is statistically identical to the wealth distribution among all society members.

The threshold p=1 marks the phase transition between ‘rich fractality’ (p<1) and ‘poor fractality’ (p>1). This threshold manifests the perfect-equality benchmark: the socioeconomic ‘ground state’ in which all society members share the very same wealth. In turn, socioeconomic inequality is gauged by quantifying deviations from the perfect-equality benchmark.

With the fractal wealth distributions described, let’s return to the PBM and PLM models. As noted above, PBM and PLM are defined by their power-law pillars. In turn, these pillars have two key parameters–the exponents δ and γ–which are both positive, and whose roles are as follows.

The exponent δ determines the pace at which the motions’ positions diffuse with time, and the pace at which the motions’ inherent fluctuations change with time. Consequently, the exponent δ determines the sub-diffusion and super-diffusion behaviors, as well as the aging and anti-aging behaviors.

For PBM, the exponent γ determines the correlations of the motion’s positions. For both PBM and PLM, the exponent γ determines the pace at which the motions’ inherent clock ticks. Also, the interplay between the exponents δ and γ determines the persistence and anti-persistence behaviors.

Mapping the axes of PBM and PLM power-laws to the axes of the power-law of fractal wealth distributions, this paper establishes Table 1. Using a single scheme–the socioeconomic classification of fractal wealth distributions–Table 1 classifies the following anomalous behaviors of PBM and PLM: sub-diffusion and super-diffusion; aging and anti-aging; sub-linear and super-linear inherent clock; and persistence and anti-persistence.

As noted above: the benchmark of the socioeconomic classification is the ground-state of perfect equality; and socioeconomic inequality is quantified via deviations from the perfect-equality benchmark. Per each anomalous behavior of PBM and PLM, Table 1 specifies (in the perfect-equality row): the PBM and PLM benchmarks that correspond to the perfect-equality benchmark. In turn, the deviations from the PBM and PLM benchmarks can be measured by socioeconomic inequality gauges–and so they will in this paper. Thus, in a nutshell, the following is stated.

▶ **This paper establishes and presents a socioeconomic-inequality perspective via which: main statistical and temporal behaviors of PBM and PLM are interpreted and scored**.

The paper is organized as follows. Gaussian-selfsimilar diffusion models are reviewed in Section 2, and the measurement of socioeconomic inequality is reviewed in Section 3. (On their own, Section 2 and Section 3 can serve readers as ‘crash introductions’ to their respective topics.) Following these reviews, the socioeconomic gauging of PBM is presented in Section 4. Elevating from the ‘Brown kingdom’ to the ‘Levy kingdom’, Section 5 reviews PLM and then presents the socioeconomic gauging of PLM. Last, Section 6 concludes with a brief recap and an outlook.

## 2. Gaussian-Selfsimilar Diffusion Models

This section reviews the prominent Gaussian diffusion models of Brownian motion (BM), fractional Brownian motion (FBM), and time-scaled Brownian motion (TSBM). Vis a vis these prominent models, power Brownian motion (PBM) is introduced and its advantages are described. The section begins with general diffusion models (Section 2.1), then it focuses on Brownian diffusion models (Section 2.2), and thereafter it focuses on PBM (Section 2.3).

### 2.1. General Diffusion Models

Along this section all random motions are considered as follows. The motions’ temporal axis is the non-negative half line t≥0, the motions’ spatial axis is the real line, and the motions initiate from the origin of the real line. Also, the motions’ positions are real-valued random variables with zero means and with finite variances.

Regarding a random motion of interest: the motion’s position at time *t* is denoted $X\left(t\right)$; and the motion’s increment over the temporal interval [a,b] is the displacement $X\left(b\right)-X\left(a\right)$. Several classes of random motions shall now be noted.

**Gaussian** class G [63,64,65]: motions whose positions–corresponding to any finite set of time points–form a random vector whose statistical distribution is multivariate Normal.
**Stationary-increments** class $I_{stat}$ [66,67,68]: motions whose increments’ statistical distributions depend only on the temporal difference b−a (rather than on the time points *a* and *b*).
**Independent-increments** class $I_{indp}$ [66,67,68]: motions whose increments’–over non-overlapping temporal intervals–are independent random variables.
**Markovian** class M [69,70,71]: motions whose future trajectory–with respect to any present time point–depends only on the present position (rather on the trajectory up to the present time point).
**Selfsimilar** class S [72,73,74]: motions whose trajectories are fractal objects that are statistically invariant to ‘zooming in’ and ‘zooming out’.

Of the above classes, the one that is universal is the selfsimilar class. Specifically, when observing from a macroscopic level a random motion that ‘runs’ on the microscopic level, then: selfsimilarity emerges universally at the macro level [8,74,75]. The elevation from the micro level to the macro level appears naturally in the context of physical diffusion processes. Indeed, physical diffusion processes take place at the micro level (of molecules) and are observed at the macro level (of humans). So, it is natural to use selfsimilar random motions as general diffusion models.

Selfsimilarity implies the variance pattern

(1) $$\mathrm{Var}\left[X\left(t\right)\right]=\mathrm{Var}\left[X\left(1\right)\right]\cdot t^{\delta }\;,$$

where δ is a positive exponent [74]. With no loss of generality, the variance at the time point one is henceforth set to be one, $\mathrm{Var}\left[X\left(1\right)\right]=1$. Thus, the variance function of Equation (1) is $\mathrm{Var}\left[X\left(t\right)\right]=t^{\delta }$.

The variance function of Equation (1) quantifies how the motion’s positions become more and more diffuse with time. Thus δ is a *diffusion exponent*, and the motion is classified as follows.

- *Sub-diffusion* when the variance function is sub-linear (δ<1).
- *Regular diffusion* when the variance function is linear (δ=1).
- *Super-diffusion* when the variance function is super-linear (δ>1).

Namely, as time progresses: in the sub-diffusion case the variance function grows infinitely slower than linear; and in the super-diffusion case the variance function grows infinitely faster than linear. The sub-diffusion and the super-diffusion cases are commonly termed *anomalous diffusion* [23,24,25,26,27,28,29,30,31,32,33,34,35].

### 2.2. Brownian Diffusion Models

Having specified several classes of random motions in Section 2.1, this subsection shall address several specific Gaussian-selfsimilar models of diffusion. These models produce random motions with continuous trajectories.

Of all random motions with continuous trajectories, the following model is arguably the best known and the most famous one.

**Brownian motion** (BM) is characterized by the intersection $G\cap I_{stat}\cap I_{indp}$ [16]. BM belongs to the Markovian class M and to the selfsimilar class S, and the BM diffusion exponent is one $\delta_{BM}=1$.

Across science and engineering, BM is the archetypal model of diffusion [4,5,16]. BM is the universal scaling limit [76,77,78] of the foundational micro-level diffusion model: the random walk [79,80,81,82,83]. As the BM diffusion exponent is one $\delta_{BM}=1$, BM is a regular diffusion. Two principal generalizations of BM that ‘upgrade’ regular diffusion to anomalous diffusion are the following.

**Fractional Brownian motion** (FBM) is characterized by the intersection $G\cap I_{stat}\cap S$ [84,85,86]. FBM does not belong to the Markovian class M, and its diffusion exponent takes values in the range $0<\delta_{FBM}\leq 2$.
**Time-scaled Brownian motion** (TSBM) is characterized by the intersection $G\cap I_{indp}\cap S$ [87,88,89]. TSBM belongs to the Markovian class M, and its diffusion exponent is positive $\delta_{TSBM}>0$.

FBM is arguably the most widely applied anomalous-diffusion generalization of BM [90,91,92,93,94,95,96,97,98,99,100,101,102,103,104,105,106,107,108,109,110]. Commonly termed “scaled BM”, TSBM is a widely applied anomalous-diffusion generalization of BM [111,112,113,114,115,116,117,118,119,120,121,122,123,124,125,126]. In this paper the term “TSBM” is used in order to distinguish this motion from another anomalous-diffusion generalization of BM: **space-scaled Brownian motion** (SSBM), which will be defined in Section 2.3.

The diffusion behaviors of FBM are coupled with its persistence behaviors [127]. Indeed, when FBM is sub-diffusive then it is *anti-persistent*, i.e. it tends to flip its direction. And when FBM is super-diffusive then it is *persistent*, i.e. it tends to maintain its direction.

The diffusion behaviors of TSBM are coupled with its aging behaviors [113,121]. Indeed, when TSBM is sub-diffusive then it is *aging*, i.e. its statistical fluctuations decay with time. And when TSBM is super-diffusive then it is *anti-aging*, i.e. its statistical fluctuations grow with time.

FBM and TSBM are attained by ‘tinkering’ with the increments’ properties of BM. On the one hand, FBM retains the stationary-increments property, and it discards the independent-increments property–thus giving rise to the persistence behaviors. On the other hand, TSBM retains the independent-increments property, and it discards the stationary-increments property–thus giving rise to the aging behaviors.

An altogether different approach to generalizing BM–rather than tinkering with the increments’ properties–is to tinker with other properties. This approach leads to the following generalization.

**Power Brownian motion** (PBM) is characterized by the intersection G∩M∩S [50,55].

From an anomalous-diffusion perspective, FBM and TSBM have–each–upsides and downsides. PBM combines together the upsides of FBM and of TSBM (while circumventing their downsides). Specifically, key anomalous-diffusion features of PBM are the following [49,50,51].

- *Diffusion*: as the TSBM diffusion exponent–and in contrast to the FBM diffusion exponent–the PBM diffusion exponent is free to assume any positive value.
- *Persistence*: as FBM–and in contrast to the TSBM–PBM displays the anomalous behaviors of persistence and anti-persistence.
- *Aging*: as TSBM–and in contrast to the FBM–PBM displays the anomalous behaviors of aging and anti-aging.

So, PBM exhibits anomalous-diffusion features that neither FBM nor TSBM can exhibit on their own. Section 2.3 shall provide further details regarding the PBM model.

### 2.3. Power Brownian Motion

The statistics of Gaussian random motions are determined by their positions’ covariances. Equivalently, the statistics of such random motions are determined by: the standard deviations of their positions; and the correlations of their positions.

When a random motion under consideration is selfsimilar then Equation (1) implies that the positions’ standard deviations are: $\mathrm{SD}\left[X\left(t\right)\right]=t^{\delta /2}$ (t≥0). And, when the random motion is PBM then the positions’ correlations are [49,50]:

(2) $$\mathrm{Cor}\left[X\left(a\right),X\left(b\right)\right]={\left(\frac{a}{b}\right)}^{\gamma /2}$$

where a≤b and b≠0, and γ is a positive *correlation exponent*.

So, the standard-deviation function and the correlation function of PBM are both power-laws. In turn, the statistics of PBM are determined by two positive parameters: the diffusion exponent δ; and the correlation exponent γ. Special cases of the PBM model are pinpointed by specific PBM exponents as follows.

- *BM*: characterized by δ=1 and γ=1.
- *Regular diffusion*: characterized by δ=1.
- *Space-scaled BM* (SSBM): characterized by γ=1.
- *Time-scaled BM* (TSBM): characterized by δ=γ.

The stochastic meaning of SSBM will be unveiled in Section 5.3. Note that when the diffusion exponent is one δ=1, and when the correlation exponent is different than one γ≠1, then: PBM yields a random motion that is a regular diffusion–yet it is *not* BM.

As in the case of TSBM, the diffusion behaviors and the aging behaviors of PBM are coupled, and they are determined by the interplay between the diffusion exponent δ and the BM diffusion exponent $\delta_{BM}=1$ [49]. The persistence behaviors of PBM are determined by the interplay between the diffusion exponent δ and the correlation exponent γ [49]. Table 2 presents a ‘map’ of the anomalous-diffusion behaviors of PBM.

PBM can be formulated as a spatio-temporal transform of BM [49,55]. Also, PBM can be formulated as the Lamperti transform of the Ornstein-Uhlenbeck process [50]. The dynamics of PBM are governed by a closed-form Markov propagator, as well as by a certain Langevin stochastic differential equation [49,50,51].

Due to these formulations and dynamics, it is easy to construct and track PBM. In sharp contrast, FBM has no such formulations and dynamics–and thus it is hard to construct and track FBM.

**PBM conclusion**. PBM is a versatile and practical Gaussian-selfsimilar model for regular and anomalous diffusion alike. PBM includes BM, as well as SSBM and TSBM, as special cases. And PBM combines together the upsides of FBM and TSBM, while circumventing their downsides. Thus, PBM is worthy of comprehensive investigation. This paper studies PBM from a socioeconomic-inequality perspective.

## 3. Measuring Socioeconomic Inequality

With regard to a given human society of interest, consider the following question: how unequal is the distribution of wealth among the society members? This question has well-established quantitative answers which will be reviewed in this section. The review starts with the notion of Lorenz curves (Section 3.1), carries on with the notion of inequality indices (Section 3.2), and culminates with fractal wealth distributions (Section 3.3 and Section 3.4).

### 3.1. Lorenz Curves

Consider a human society comprising of members, where each member has a (non-negative) wealth. The members are ordered from the poorest (with the smallest wealth) to the richest (with the largest wealth). The distribution of wealth among the society members is quantified–in a universally calibrated way–by *Lorenz curves* [128,129,130,131].

The Lorenz curves come in pairs [132,133]: a ‘bottom-up’ curve $y=L_{BU}\left(x\right)$; and a ‘top-down’ curve $y=L_{TD}\left(x\right)$. Both Lorenz curves reside in the unit square 0≤x,y≤1, and they are monotone increasing from the square’s south-west corner (x=y=0) to the north-east corner (x=y=1).

The bottom-up Lorenz curve $y=L_{BU}\left(x\right)$ is convex, and its socioeconomic definition is [132,133]: the low 100x% of the poorest society members hold, collectively, 100y% of the society’s overall wealth. The top-down Lorenz curve $y=L_{TD}\left(x\right)$ is concave, and its socioeconomic definition is [132,133]: the top 100x% of the richest society members hold, collectively, 100y% of the society’s overall wealth.

[More specifically, consider the society to comprise of *n* members, with wealths $w_{1}\leq w_{2}\leq \cdots \leq w_{n}$. Then, the society’s overall wealth is $w=w_{1}+\cdots +w_{n}$. The values of the Lorenz curves at the grid points x=k/n (where k=1,2,⋯,n) are: $L_{BU}\left(k/n\right)=\left(w_{1}+\cdots +w_{k}\right)/w$; and $L_{TD}\left(k/n\right)=\left(w_{n-k+1}+\cdots +w_{n}\right)/w$. The grid values are extended to all values by linear extrapolation.]

Due to their definitions, the two Lorenz curves are coupled by the relation

(3) $$L_{BU}\left(x\right)+L_{TD}\left(1-x\right)=1\;.$$

So, the bottom-up Lorenz curve and the top-down Lorenz curve manifest two alternative ways of coding the very same information. And, using Equation (3) one can easily shift from each Lorenz curve to the other.

Due to their shapes, the Lorenz curves are ‘ordered’ as follows:

(4) $$0\leq L_{BU}\left(x\right)\leq x\leq L_{TD}\left(x\right)\leq 1\;.$$

Namely, the bottom-up Lorenz curve $y=L_{BU}\left(x\right)$ is bounded from below by the value zero, and is bounded from above by the diagonal line y=x. And, the top-down Lorenz curve $y=L_{TD}\left(x\right)$ is bounded from below by the diagonal line y=x, and is bounded from above by the value one. These bounds manifest two antithetical socioeconomic poles.

One pole is the socioeconomic ‘ground state’ of *perfect equality*: all society members have exactly the same (positive) wealth. This state is defined by the diagonal line y=x, and it is attained if and only if the two Lorenz curves coincide, $L_{BU}\left(x\right)=L_{TD}\left(x\right)$.

The other pole is the socioeconomic state of *perfect inequality*: a zero fraction of the society members hold, collectively, the society’s entire wealth; and all other society members have zero wealth. This state is attained when the society’s population grows infinitely large, and if and only if: the bottom-up Lorenz curve coincides with its lower-bound $L_{BU}\left(x\right)=0$ (where 0≤x<1); and the top-down Lorenz curve coincides with its upper bound $L_{TD}\left(x\right)=1$ (where 0<x≤1).

### 3.2. Inequality Indices

The pair of Lorenz curves defines a Lorenz set [132,133]. Namely, the Lorenz set comprises of the unit-square points that reside between the two Lorenz curves [132,133]:

(5) $$L=\left\{0\leq x,y\leq 1|L_{BU}\left(x\right)\leq y\leq L_{TD}\left(x\right)\right\}\;.$$

The Lorenz set provides a quantitative geometric description of the distribution of wealth among the society members.

The ‘minimal’ Lorenz set is the diagonal line $L_{pe}=\left\{0\leq x,y\leq 1|x=y\right\}$, which characterizes the socioeconomic ‘ground state’ of perfect equality. The ‘maximal’ Lorenz set is the unit square $L_{pi}=\left\{0\leq x,y\leq 1\right\}$, which characterizes the socioeconomic state of perfect inequality. These minimal and maximal sets are, respectively, the lower and upper ‘bounds’ of the Lorenz set: $L_{pe}\subseteq L\subseteq L_{pi}$.

Inequality indices score, numerically, the degree of socioeconomic inequality in human societies [56,57,58,59,60]. An inequality index is a functional of the Lorenz set $I\left(L\right)$ that takes values in the unit interval $0\leq I\left(L\right)\leq 1$, and that satisfies the following properties [132,133].

- *Zero score*. The inequality index yields the minimal score if and only if the Lorenz set is the minimal one: $L=L_{pe}\Leftrightarrow I\left(L\right)=0$.
- *Unit score*. The inequality index yields the maximal score when the Lorenz set is the maximal one: $L=L_{pi}\Rightarrow I\left(L\right)=1$.
- *Monotonicity*. The inequality index is a monotone increasing functional of its Lorenz-set ‘variable’: $L_{1}\subseteq L_{2}\Rightarrow I\left(L_{1}\right)\leq I\left(L_{2}\right)$.

So, the inequality-index score is zero only when the socioeconomic state is that of perfect equality, and it is one when the socioeconomic state is that of perfect inequality. The smaller the score–the more equal the distribution of wealth among the society members, and the more egalitarian the society. Conversely, the larger the score–the less equal the distribution of wealth among the society members, and the less egalitarian the society.

There are many approaches to devise inequality indices. Three such approaches–leading to five different inequality indices–shall now be noted. The resulting inequality indices are detailed in Table 3.

The first approach is the area of the Lorenz set. This approach yields the *Gini index* $I_{Gini}$, which is arguably the most popular and the most widely applied socioeconomic inequality index [134,135,136,137,138]. In addition to its Lorenz-set representation, the Gini index has many more representations [139,140]–each interpreting and illuminating the Gini index from a different ‘angle’.

The second approach is the diameter of the Lorenz set [59,132,133]. From a ‘vertical view’: the diameter is the maximal vertical distance between the pair of Lorenz curves, this maximal distance is attained along the line $x=\frac{1}{2}$, and it yields the *vertical-diameter index* $I_{VD}$. From a ‘horizontal view’: the diameter is the maximal horizontal distance between the pair of Lorenz curves, this maximal distance is attained along the line $y=\frac{1}{2}$, and it yields the *horizontal-diameter index* $I_{HD}$.

The third approach can be perceived as a combination of the first and second approaches, and it yields two indices [141]: the *vertical-Bonferroni index* $I_{VB}$, which is the ‘classic’ Bonferroni index [142,143,144,145,146,147,148,149]; and the *horizontal-Bonferroni index* $I_{HB}$. These indices are functionals of the Lorenz curves, and in addition to their Lorenz-curves representations, they have many more representations [141]–each interpreting and illuminating the Bonferroni indices from a different ‘angle’.

### 3.3. Fractal Wealth Distributions

There are infinitely many ways of distributing wealth among the members of a given human society. Of these many ways, there are two particular ways in which wealth can be distributed in a ‘fractal’ fashion [61,62]: *rich fractality* and *poor fractality*. These fractal wealth distributions shall now be described and quantified.

Qualitatively, rich fractality holds when the distribution of wealth among the rich is identical to the distribution of wealth among the society’s entire population. Quantitatively, rich fractality is characterized by the top-down Lorenz curve $L_{TD}\left(x\right)=x^{p}$ [61,62], where 0<p<1.

Qualitatively, poor fractality holds when the distribution of wealth among the poor is identical to the distribution of wealth among the society’s entire population. Quantitatively, poor fractality is characterized by the bottom-up Lorenz curve $L_{BU}\left(x\right)=x^{p}$ [61,62], where 1<p<∞.

Consider the power-law Lorenz curve

(6) $$L\left(x\right)=x^{p}\;,$$

where the power *p* is positive. As described in Section 3.1: Lorenz curves come in pairs, and the pairs are coupled by Equation (3). Hence, the ‘twin’ of the Lorenz curve of Equation (6) is the Lorenz curve $1-L\left(1-x\right)=1-{\left(1-x\right)}^{p}.$

The power-law Lorenz curve of Equation (6), as well as its twin Lorenz curve, characterize–via their positive power parameter *p*–the three following socioeconomic regimes.

- *Rich fractality* when p<1 (in which case the Lorenz curve of Equation (6) is top-down).
- *Perfect equality* when p=1 (in which case the Lorenz curve of Equation (6) is both top-down and bottom-up).
- *Poor fractality* when p>1 (in which case the Lorenz curve of Equation (6) is bottom-up).

So, the socioeconomic state of perfect equality is the borderline between rich fractality and poor fractality. The antithetical socioeconomic state of perfect inequality is attained at the following ‘fractal extremes’: the limit p→0 of rich fractality; and the limit p→∞ of poor fractality.

As the power *p* crosses the threshold one, the shape of the power-law Lorenz curve of Equation (6) changes from concave (p<1) to convex (p>1). From a socioeconomic perspective–as described below–the threshold p=1 manifests a phase transition.

With regard to a society whose wealth distribution is quantified by the power-law Lorenz curve of Equation (6): sample at random a member of the society, and set *W* to be the wealth of this member. Also, with no loss of generality, consider the average wealth (of the society members) to be one. Then, the random variable *W* has mean one (E[W]=1), and its statistics are determined by the power *p* as follows.

- *Rich fractality*: *W* takes values in the range (p,∞), and its statistical distribution is *Pareto*.
- *Perfect equality*: *W* is a *deterministic* random variable.
- *Poor fractality*: *W* takes values in the range (0,p), and its statistical distribution is *inverted Pareto*.

So, indeed, the threshold p=1 marks a profound phase transition. The Pareto statistical distributions [150] of the random variable *W* (below and above the threshold p=1) are specified in Table 4.

The derivations of the Pareto statistical distributions (of Table 4) are detailed in [61,62], and are based on the Pietra representations of Lorenz curves [151]: formulae that connect Lorenz curves on the one hand, and statistical distribution functions on the other hand. In short, these derivations are described as follows.

Denote by $F\left(w\right)=\mathrm{Pr}\left(W\leq w\right)$ (0<w<∞) the cumulative distribution function of the random variable *W*. When the Lorenz curve is bottom-up then the Pietra representation implies that: the derivative of the Lorenz curve is the inverse of the cumulative distribution function, $L^{\prime }\left(x\right)=F^{-1}\left(x\right)$.

Denote by $\bar{F}\left(w\right)=\mathrm{Pr}\left(W>w\right)$ (0<w<∞) the tail distribution function of the random variable *W*. When the Lorenz curve is top-down then the Pietra representation implies that: the derivative of the Lorenz curve is the inverse of the tail distribution function, $L^{\prime }\left(x\right)={\bar{F}}^{-1}\left(x\right)$.

The relations $L^{\prime }\left(x\right)=F^{-1}\left(x\right)$ and $L^{\prime }\left(x\right)={\bar{F}}^{-1}\left(x\right)$ hold for general Lorenz curves, and for general distribution functions with a unit mean. In particular, for the power-law Lorenz curve of Equation (6) these relations (and a bit of algebra) yield Table 4.

### 3.4. Fractal Inequality

As noted at the opening of this section, the section’s pivotal question is the following. With regards a given human society of interest: how unequal is the distribution of wealth among the society members? Considering the distribution of wealth to be fractal, the society’s Lorenz curve is that of Equation (6), and it is parameterized by the positive power *p*.

In turn, a straightforward answer to the inequality question is: the deviation of the power *p* from the threshold one, |p−1|. Indeed, the absolute deviation |p−1| is arguably the simplest answer that comes in mind. However, this answer has drawbacks.

On the one hand, the absolute-deviation answer yields markedly asymmetric values: in the rich-fractality regime (p<1) the values are bounded, 0<|p−1|<1; whereas in the poor-fractality regime (p>1) the values are unbounded, 0<|p−1|<∞. On the other hand, the absolute-deviation answer is intuitive, and does not ‘stand’ on a socioeconomic foundation.

Inequality indices–which were described in Section 3.2–have no such drawbacks. Applying the inequality indices of Section 3.2 to the power-law Lorenz curve of Equation (6) yields ‘fractal outcomes’ that are detailed in the right column of Table 3. These fractal outcomes follow via straightforward calculations using the general formulae of Table 3, and they are discussed below.

When p<1 then the fractal horizontal-Bonferroni index is $I_{HB}=1-p$. And when p>1 then the fractal vertical-Bonferroni index is $I_{VB}=1-1/p$. These fractal outcomes can be combined to the following ‘amalgam’: the fractal Bonferroni index $I_{Bonf}=1-\min\left\{p,1/p\right\}$.

When p<1 then the fractal vertical-diameter index is $I_{VD}=2^{1-p}-1$. And when p>1 then the fractal horizontal-diameter index is $I_{HD}=2^{1-1/p}-1$. These fractal outcomes can be combined to the following ‘amalgam’: the fractal diameter index $I_{Diam}=2^{1-\min\{p,1/p\}}-1$.

Note that the fractal diameter index is, in effect, an exponential version of the fractal Bonferroni index: $I_{Diam}=2^{I_{Bonf}}-1$. Also note that the fractal Bonferroni index $I_{Bonf}$ and the fractal diameter index $I_{Diam}$, as well as the fractal Gini index $I_{Gini}=\left|p-1\right|/\left(p+1\right)$, share the following property: invariance with respect to the parameter transformation p↔1/p–which is a one-to-one and onto mapping between rich fractality and poor fractality.

Shifting from the power parameter *p* to its logarithm l=ln(p), the reciprocal transformation p↔1/p shifts to the mirroring transformation l↔−l. In turn, symmetry between rich fractality and poor fractality emerges.

Indeed, negative values of the logarithmic parameter l<0 characterize rich fractality, and positive values of the logarithmic parameter l>0 characterize poor fractality. And, the following holds for any fractal inequality index with the above invariance property: for any positive number *n*, the inequality score of rich fractality with parameter l=−n is the same as the inequality score of poor fractality with parameter l=n.

Stripping the Gini-index outcome off its absolute value yields the fractal Gini gauge

(7) $$G=\frac{p-1}{p+1}=\tanh\left(\frac{1}{2}l\right)\;.$$

The hyperbolic-tangent representation of the fractal Gini gauge, $g\left(l\right)=\tanh\left(\frac{1}{2}l\right)$ (−∞<l<∞), has a sigmoidal shape. The details of the sigmoidal shape are as follows.

The hyperbolic tangent is monotone increasing from the lower-bound value $\lim_{l\to -\infty }g\left(l\right)=-1$, via the value $g\left(0\right)=0$, to the upper-bound value $\lim_{l\to \infty }g\left(l\right)=+1$.
Negative values $g\left(l\right)<0$ characterize rich fractality, and the lower-bound value −1 characterizes the perfect-inequality extreme of rich fractality.
Positive values $g\left(l\right)>0$ characterize poor fractality, and the upper-bound value +1 characterizes the perfect-inequality extreme of poor fractality.
The zero value $g\left(0\right)=0$ characterizes perfect equality, and is the inflection point of the hyperbolic tangent–which is convex when negative, and which is concave when positive.

## 4. Socioeconomic Gauging of Power Brownian Motion

Having reviewed power Brownian motion (PBM) in Section 2, and having reviewed the measurement of socioeconomic inequality in Section 3, the stage is set to apply the latter to the former. Namely, the stage is set for the socioeconomic gauging of PBM.

The socioeconomic gauging is established and presented via a temporal approach involving three PBM clocks (Section 4.1). Then, the temporal approach is discussed (Section 4.2), and a complementary statistical approach is presented (Section 4.3).

Some readers may find the statistical approach (of Section 4.3) to be more straightforward than the temporal approach (of Section 4.1). However, the temporal approach is advantageous due to the following facts.

The temporal approach uses a single perspective–that of a power-law clock–and applies this one perspective to the various anomalous behaviors of PBM. In contrast, the statistical approach uses three different perspectives–one per each of the anomalous behaviors (that are addressed in Section 4.2). More importantly, when elevating from PBM to power Levy motion (as will be shown in Section 5): the temporal approach carries on ‘as is’, whereas the statistical approach does not.

As an illustrative metaphor, envisage a hotel with two residential floors: a Brown floor; and a Levy floor. On the one hand, the temporal approach is akin to a master key that opens all doors on both the Brown and Levy floors. On the other hand, the statistical approach is akin to a set of keys (a key per door) that open the doors on the Brown floor alone.

### 4.1. PBM Clocks

In this subsection PBM is tracked along the temporal window [0,T], where *T* is a fixed and positive time point. Thus, the temporal variable *t* takes values in the range 0≤t≤T.

Consider a power-law clock $C\left(t\right)=t^{p}$, where *p* is a positive power. Measuring the clock’s count at the time point *t*–relative to the clock’s count at the time point *T*–yields the clock ratio $C\left(t\right)/C\left(T\right)={\left(t/T\right)}^{p}$. The temporal ratio r=t/T manifests the relative location of the time point *t* within the temporal window [0,T]. In terms of the temporal ratio *r*, the clock ratio is the power-law $r^{p}$ (0≤r≤1).

Now, map the axes that underpin the clock ratio $r^{p}$ to the axes that underpin Lorenz curves. This mapping yields the power-law Lorenz curve of Equation (6). In turn, the following matching–between a clock classification on the one hand, and the socioeconomic regimes of Section 3.3 on the other hand–is attained.

- *Sub-linear* clock (p<1) corresponds to *rich fractality*.
- *Linear* clock (p=1) corresponds to *perfect equality*.
- *Super-linear* clock (p>1) corresponds to *poor fractality*.

The clock classification is based on a comparison of the subjective clock $C\left(t\right)$ to the objective clock *t*. Indeed, as time progresses: in the sub-linear case the subjective clock grows infinitely slower than the objective clock; and in the super-linear case the subjective clock grows infinitely faster than the objective clock.

As described in Section 2.1 with regard to a general selfsimilar random motion: the motion’s variance function is $\mathrm{Var}\left[X\left(t\right)\right]=t^{\delta }$, where δ is the positive diffusion exponent. In effect, this variance function manifests the motion’s ‘diffusion clock’ $C_{dif}\left(t\right)=t^{\delta }$. Namely, the diffusion clock counts time according to the pace at which the motion’s positions diffuse.

As shall be described in Section 5.3: the inherent clock of PBM is $C_{inh}\left(t\right)=t^{\gamma }$, where γ is the positive correlation exponent. In terms of its diffusion clock, the inherent clock of PBM admits the representation $C_{inh}\left(t\right)={\left[C_{dif}\left(t\right)\right]}^{\gamma /\delta }$. This representation yields the relative clock $C_{rel}\left(t\right)=t^{\gamma /\delta }$. Namely, the relative clock counts the PBM inherent time according to the ticks of the PBM diffusion clock.

So, three power-law clocks emanate from PBM: a diffusion clock whose power is the diffusion exponent p=δ; an inherent clock whose power is the correlation exponent p=γ; and a relative clock whose power is the ratio of the correlation exponent to the diffusion exponent p=γ/δ. The above clock classification, together with its socioeconomic match, holds for each of these clocks.

The attained clock classifications are showcased–vis a vis the PBM anomalous behaviors–in rows #1 to #3 of Table 5. A discussion of the clock classifications follows in Section 4.2.

### 4.2. Discussion

Main anomalous behaviors of general diffusion models (as noted in Section 2.1) are: sub-diffusion and super-diffusion; aging and anti-aging; and persistence and anti-persistence. As described in Section 2.1 with regard to selfsimilar diffusion models: the diffusion behaviors are determined by the positive diffusion exponent δ.

As noted in Section 2.2, PBM is the only selfsimilar diffusion model that is Gaussian and Markovian. In addition to its diffusion exponent δ–which is shared by all selfsimilar diffusion models–PBM has the positive exponent γ. As described in Section 2.3, γ is a correlation exponent that determines the correlation structure of the PBM positions. And, as noted in Section 4.1 (and as will be shown in Section 5.3), γ also determines the inherent clock of PBM.

As described in Section 2.3: the aging behaviors of PBM are coupled to the diffusion behaviors (which are determined by the diffusion exponent δ); and the persistence behaviors of PBM are determined by the interplay between the diffusion exponent δ and the clock exponent γ. Table 2 presented a ‘map’ of the PBM anomalous behaviors.

Section 4.1 showcased three different PBM clocks. Per each of these clocks: Section 4.1 established a clock classification and a matching socioeconomic classification–whose benchmark is the socioeconomic ‘ground state’ of perfect equality. These classifications are summarized in rows #1 to #3 of Table 5, and they provide **a socioeconomic-inequality perspective via which the PBM anomalous behaviors are interpreted and scored**.

Indeed, the classification of the PBM diffusion clock is in accord with the PBM diffusion and aging behaviors–for which the benchmark is regular diffusion (δ=1). The classification of the PBM relative clock is in accord with the PBM persistence behaviors–for which the benchmark is TSBM (γ=δ). And, the benchmark for the PBM inherent clock is SSBM (γ=1). These benchmarks are highlighted in row #2 of Table 5.

As described in Section 3.2, socioeconomic deviations from the benchmark of perfect equality are scored by inequality indices. In particular, with regard to fractal wealth distributions: Section 3.4 presented several such inequality indices, as well as the fractal Gini gauge of Equation (7). Per each of the PBM clocks: row #4 of Table 5 specifies the corresponding fractal Gini gauge.

The fractal Gini gauges in row #4 of Table 5 provide numerical scores–with values ranging between −1 and +1–that quantify the deviation of PBM from its benchmarks.

- A gauge based on the diffusion exponent δ, which scores the deviation from regular-diffusion.
- A gauge based on the correlation exponent γ, which scores the deviation from SSBM.
- A gauge based on both exponents, δ and γ, which scores the deviation from TSBM.

Note that PBM is BM if and only if the following condition holds: any two of the three scores is zero. In conclusion, and ‘in a nutshell’, the following is stated.

▶ **Inequality indices in general, and the fractal Gini gauge in particular, provide quantitative answers to the question: what is the ‘degree of anomaly’ of each of the PBM anomalous behaviors?**

Theoretically, the quantitative answers are ‘solid’–as they ‘stand’ on a well-established socioeconomic foundation (described in Section 3). Practically, the quantitative answers are easy to use and comprehend. Indeed–as evident from the right column of Table 3, and from row #4 of Table 5–these answers are given by markedly simple and transparent formulae.

The degrees of anomaly can be measured directly by the PBM exponents as follows. Regarding the anomaly relative to the regular-diffusion benchmark (δ=1): directly by the diffusion exponent δ, via the absolute deviation |δ−1|. Regarding the anomaly relative to the SSBM benchmark (γ=1): directly by the correlation exponent γ, via the absolute deviation |γ−1|. And regarding the anomaly relative to the TSBM benchmark (γ=δ): directly by the exponents δ and γ, via the absolute deviation |γ−δ|.

These absolute deviations are identical–in both their essence and their form–to the absolute-deviation answer that was described at the opening of Section 3.4 (regarding the socioeconomic inequality of fractal wealth distributions, measured relative to the perfect-equality benchmark). Section 3.4 described the drawbacks of using the absolute-deviation answer–in contrast to the advantages of using inequality indices. This argumentation applies ‘as is’ to using the above absolute deviations–in contrast to the advantages of using the quantitative answers of the above statement.

### 4.3. Statistical Approach

The results showcased in Table 5 are based on the three PBM clocks: the diffusion clock, the inherent clock, and the relative clock. As described in Section 4.1, the clock ratio of the diffusion clock is the following variance ratio:

(8) $$\frac{\mathrm{Var}[X\left(t\right)]}{\mathrm{Var}[X\left(T\right)]}=r^{\delta }\;,$$

where r=t/T.

Mapping the axes that underpin the right-hand side of Equation (8) to the axes that underpin Lorenz curves yields the power-law Lorenz curve of Equation (6)–with power p=δ. So, the socioeconomic match of the diffusion-clock classification (see Table 5) is attained.

This subsection shall present statistical formulae that–akin to the statistical formula of Equation (8), which leads to the socioeconomic match of the diffusion-clock classification–lead to the socioeconomic matches of the inherent-clock and the relative-clock classifications. To that end the temporal ratio r=t/T will be used, as well as the following result regarding the bivariate Normal distribution [63,64,65].

Consider a two-dimensional random vector $\left(Z_{1},Z_{2}\right)$ whose statistical distribution is bivariate Normal with the following characteristics: zero means $E[Z_{1}]=0$ and $E[Z_{2}]=0$; positive variances $\mathrm{Var}\left[Z_{1}\right]=v_{1}$ and $\mathrm{Var}\left[Z_{2}\right]=v_{2}$; and correlation $\mathrm{Cor}[Z_{1},Z_{2}]=\rho$. Then, the conditional statistical distribution of $Z_{2}$–given the information $Z_{1}$–is Normal with the following characteristics: conditional mean $E\left[Z_{2}|Z_{1}\right]=\rho \sqrt{v_{2}/v_{1}}\cdot Z_{1}$; and conditional variance $\mathrm{Var}\left[Z_{2}|Z_{1}\right]=\left(1-\rho^{2}\right)v_{2}$.

#### 4.3.1. Inherent Clock

Apply the conditional variance formula $\mathrm{Var}\left[Z_{2}|Z_{1}\right]=\left(1-\rho^{2}\right)v_{2}$ to the random variables $Z_{1}=X\left(t\right)$ and $Z_{2}=X\left(T\right)$. Using Equations (1) and (2), and doing a bit of algebra, yields

(9) $$\frac{\mathrm{Var}\left[X\left(T\right)\right]-\mathrm{Var}\left[X\left(T\right)|X\left(t\right)\right]}{\mathrm{Var}[X\left(T\right)]}=r^{\gamma }\;.$$

The left-hand side of Equation (9) manifests the following quantity: the variance reduction of the position $X\left(T\right)$ given the information $X\left(t\right)$ (the enumerator of the left-hand side), measured relative to the variance of the position $X\left(T\right)$ (the denominator of the left-hand side).

Mapping the axes that underpin the right-hand side of Equation (9) to the axes that underpin Lorenz curves yields the power-law Lorenz curve of Equation (6)–with power p=γ. So, the socioeconomic match of the inherent-clock classification (see Table 5) is attained.

An alternative formulation of Equation (9) is

(10) $$\frac{\mathrm{Var}[X\left(T\right)|X\left(t\right)]}{\mathrm{Var}[X\left(T\right)]}=1-r^{\gamma }\;.$$

The left-hand side of Equation (10) manifests the following quantity: the variance of the position $X\left(T\right)$ given the information $X\left(t\right)$ (the enumerator of the left-hand side), measured relative to the variance of the position $X\left(T\right)$ (the denominator of the left-hand side).

The temporal ratio $\bar{r}=\left(T-t\right)/T$ manifests the distance of the time point *t* from the time point *T*, measured relative to the length of the temporal window [0,T]. In terms of the temporal ratio $\bar{r}$, the right-hand side of Equation (10) is the curve $1-{\left(1-\bar{r}\right)}^{\gamma }$ ($0\leq \bar{r}\leq 1$).

Mapping the axes that underpin the curve $1-{\left(1-\bar{r}\right)}^{\gamma }$ to the axes that underpin Lorenz curves yields the ‘twin’ of the power-law Lorenz curve of Equation (6)–with power p=γ. So, again, the socioeconomic match of the inherent-clock classification (see Table 5) is attained.

#### 4.3.2. Relative Clock

Apply the conditional mean formula $E\left[Z_{2}|Z_{1}\right]=\rho \sqrt{v_{2}/v_{1}}\cdot Z_{1}$ to the random variables $Z_{1}=X\left(t\right)$ and $Z_{2}=X\left(T\right)$. Using Equations (1) and (2), and doing a bit of algebra, yields

(11) $$E\left[X\left(T\right)-X\left(t\right)|X\left(t\right)\right]=-\underset{\mathrm{slope}}{\underset{︸}{\frac{r^{\delta /2}-r^{\gamma /2}}{r^{\delta /2}}}}\cdot X\left(t\right)\;.$$

The left-hand side of Equation (11) manifests the following quantity: the mean of the increment $X\left(T\right)-X\left(t\right)$ given the information $X\left(t\right)$.

The right-hand side of Equation (11) is a linear function of the given information–the position $X\left(t\right)$. Applying a square root to Equation (8) yields the standard-deviation ratio $\sqrt{v_{1}/v_{2}}=r^{\delta /2}$. In terms of the quantity $u=r^{\delta /2}$ the slope appearing on the right-hand side of Equation (11) is $(u-u^{\gamma /\delta })/u$.

Observing the integrands of the general Bonferroni formulae of Table 3, note that: these integrands are identical to the slope $(u-u^{\gamma /\delta })/u$ if and only if the Lorenz curve is the power-law of Equation (6), with power p=γ/δ. Thus, mapping the axes that underpin the ‘slope term’ $u^{\gamma /\delta }$ to the axes that underpin Lorenz curves yields the power-law Lorenz curve of Equation (6)–with power p=γ/δ. In turn, the socioeconomic match of the relative-clock classification (see Table 5) is attained.

## 5. From Brown to Levy

So far, this paper addressed PBM. Elevating from the ‘Brown kingdom’ to the ‘Levy kingdom’, this section addresses power Levy motion (PLM) [52,53,54]. To that end the section reviews the following topics: the symmetric Levy-stable distribution and motion (Section 5.1 and Section 5.2); and PLM and its Markovian propagator (Section 5.3 and Section 5.4). Then, the socioeconomic gauging of PLM is presented (Section 5.5).

### 5.1. Symmetric Levy-Stable Distribution

Consider a real-valued random variable *R*. The statistical distribution of the random variable is *symmetric Levy-stable* (SLS) [152,153,154] when its Fourier transform admits the form

(12) $$E\left[\exp\left(i\theta \frac{R-m}{s}\right)\right]=\exp\left(-\frac{1}{\lambda }{\left|\theta \right|}^{\lambda }\right)\;,$$

where −∞<θ<∞ is the Fourier variable. The three parameters appearing in Equation (12)–*m*, *s*, and λ–are as follows.

The parameter *m* is real valued, it is the median of the SLS distribution, and it manifests the distribution’s ‘center’. The parameter *s* is positive valued, it is the scale of the SLS distribution, and it manifests the distribution’s ‘width’ about its center. The parameter λ–termed *Levy exponent*–takes values in the range 0<λ≤2, and it determines the statistical behavior of the SLS distribution.

When 0<λ≤1 then the mean absolute deviation of the SLS distribution from its center is infinite E[|R−m|]=∞, and hence the distribution does not have a well-defined mean. When 1<λ≤2 then the distribution’s mean is its median E[R]=m, and the distribution’s variance is as follows: infinite Var[R]=∞ when 1<λ<2; and finite $\mathrm{Var}\left[R\right]=s^{2}$ when λ=2.

In fact, when λ=2 then the SLS distribution reduces to the Normal distribution–with mean *m* and with variance $s^{2}$. In general, i.e. for all values 0<λ≤2 of the Levy exponent: the quantity $s^{\lambda }$ manifests an *extended variance* of the SLS distribution [155].

### 5.2. Symmetric Levy-Stable Motion

Akin to Section 2.1, consider random motions that are as follows. The motions’ temporal axis is the non-negative half line t≥0, the motions’ spatial axis is the real line, and the motions initiate from the origin of the real line. In the context of such random motions, the following class is foundational.

**Levy** class $I_{stat}\cap I_{indp}$ [66,67,68]: motions that display the stationary-increments property and the independent-increments property (both described in Section 2.1).

Further consider random motions whose positions are symmetric real-valued random variables. For such random motions the positions’ medians are zero (and when the positions’ means are well-defined then they are also zero). In the context of such random motions, the following Levy motion is of principal importance.

**SLS motion** is characterized by the intersection $I_{stat}\cap I_{indp}\cap S$.

The name “SLS motion” is due to the fact that the statistical distributions of this motion’s positions are SLS [66,67,68]. Indeed, denote by $L\left(t\right)$ the position of the SLS motion at the time point *t*. Then, the Fourier transform of the motion’s positions is

(13) $$E\left\{\exp\left[i\theta L\left(t\right)\right]\right\}=\exp\left(-\frac{1}{\lambda }{\left|\theta \right|}^{\lambda }\cdot t\right)$$

(−∞<θ<∞). Namely, the statistical distribution of the position $L\left(t\right)$ is SLS with the following parameters: median zero; and scale $t^{1/\lambda }$.

When λ=2 then the SLS motion is BM, and the temporal term *t* appearing on the right-hand side of Equation (13) is the variance of the BM positions. In general, i.e. for all values 0<λ≤2 of the Levy exponent: the temporal term *t* appearing on the right-hand side of Equation (13) manifests the *extended variance* of the SLS-motion positions [155].

Stochastic models based on the SLS motion attracted major interest in science and engineering [156,157,158,159,160,161,162,163,164,165,166,167]. To date, such models are continuing to attract much interest [168,169,170,171,172,173,174,175,176,177,178,179,180,181,182,183,184,185,186,187,188,189,190].

### 5.3. Power Levy Motion

*Power Levy motion* (PLM) is a spatio-temporal transformation of the SLS motion. Specifically, the PLM positions are attained via the following spatio-temporal transformation of the SLS-motion positions [52,53]:

(14) $$X\left(t\right)=t^{\frac{\delta -\gamma }{\lambda }}L\left(t^{\gamma }\right)\;,$$

where γ and δ are positive parameters of the transformation. (As above, λ is the Levy exponent of the SLS motion.)

The temporal power-law $t^{\gamma }$ appearing on the right-hand side of Equation (14) is, in effect, the inherent ‘clock’ of PLM. Thus, the parameter γ is the *clock exponent* of PLM.

Equations (13) and (14) imply that the Fourier transform of the PLM positions is

(15) $$E\left\{\exp\left[i\theta X\left(t\right)\right]\right\}=\exp\left(-\frac{1}{\lambda }{\left|\theta \right|}^{\lambda }\cdot t^{\delta }\right)$$

(−∞<θ<∞). The temporal power-law $t^{\delta }$ appearing on the right-hand side of Equation (15) manifests the extended variance of the PLM positions.

The PLM extended variance $t^{\delta }$ quantifies how the PLM positions become more and more diffuse with time. Thus, the parameter δ is the *diffusion exponent* of PLM.

Special cases of the PLM model are pinpointed by specific PLM exponents as follows.

- *SLS motion*: $X\left(t\right)=L\left(t\right)$, characterized by δ=1 and γ=1.
- *Space-scaled SLS motion*: $X\left(t\right)=t^{(\delta -1)/\lambda }\cdot L\left(t\right)$, characterized by γ=1.
- *Time-scaled SLS motion*: $X\left(t\right)=L\left(t^{\gamma }\right)$, characterized by γ=δ.

As noted in Section 5.2, when λ=2 then the SLS motion is BM. Consequently, when λ=2 then [52,53]: PLM is PBM; the special space-scaled case is SSBM; and the special time-scaled case is TSBM.

So, in particular, Equation (14) provides a formulation of PBM as a spatio-temporal transformation of BM. In turn, the above special cases provide formulations of SSBM and of TSBM as spatio-temporal transformations of BM.

### 5.4. Markovian Propagator

Due to their independent-increments property, Levy motions in general–and hence the SLS motion in particular–belong to the Markovian class M [66,67,68]. The spatio-temporal transformation of Equation (14) maps the SLS-motion input to the PLM output. Due to its spatio-temporal structure, the transformation of Equation (14) maps Markovian inputs to Markovian outputs. So, as the SLS motion, also PLM belongs to the Markovian class M.

The evolution of a general Markovian motion is governed by its *propagator* [69,70,71]: a statistical rule that specifies how to advance from the motion’s (observed) position at a present time point to the motion’s position at any future time point. The PLM propagator follows from the statistical properties of the SLS motion, and from the spatio-temporal transformation of Equation (14) [52,53].

Consider PLM and two positive time points: the present time point *t* and the future time point *T* (where t<T). Then, the conditional statistical distribution of the future position $X\left(T\right)$–given the present position $X\left(t\right)$–is SLS with Levy exponent λ. In turn, so is the conditional statistical distribution of the increment $X\left(T\right)-X\left(t\right)$. The parameters of these conditional SLS distributions are detailed in Table 6.

### 5.5. Socioeconomic Gauging of Power Levy Motion

The Normal distribution is characterized by its pair of parameters: the mean, which manifests the distribution’s ‘center’; and the variance–the square of the standard deviation, which manifests the distribution’s ‘width’ about its center. Elevating from the Normal distribution to the SLS distribution, the pair of the Normal parameters are ‘elevated’ as follows: mean ↦ median; variance ↦ extended variance.

In perfect accord with the elevation from the Normal distribution to the SLS distribution is: the elevation from BM to the SLS motion; and, in turn, the elevation from PBM to PLM. The spatio-temporal transformation that maps the SLS motion to PLM (and, in particular, BM to PBM) has two positive parameters: the diffusion exponent δ; and the clock exponent γ.

The variance function of Equation (1) quantifies the diffusivity, as time progresses, of the PBM positions. In turn, as described in Section 4.1: the variance function of Equation (1) is the ‘diffusion clock’ of PBM. Elevating from PBM to PLM, the extended variance of the PLM positions is the same as the variance of the PBM positions. So, the PLM has the same ‘diffusion clock’ as PBM: $C_{dif}\left(t\right)=t^{\delta }$.

As evident from Equation (14), the ‘inherent clock’ of PLM is $C_{inh}\left(t\right)=t^{\gamma }$. Consequently, as explained in Section 4.1: representing the inherent clock $C_{inh}\left(t\right)=t^{\gamma }$ in terms of the diffusion clock $C_{dif}\left(t\right)=t^{\delta }$ yields the relative clock $C_{rel}\left(t\right)=t^{\gamma /\delta }$. So, PBM and PLM share the same triplet of clocks, and hence: the clock classifications–which were established and described Section 4.1–hold for PLM just as they hold for PBM.

The anomalous behaviors of PBM were reviewed in Section 2, and were summarized in Table 2. As established in [52,53], Table 2 holds also for PLM–after extending the following notions from the ‘Brown kingdom’ to the ‘Levy kingdom’: sub-diffusion and super-diffusion; persistence and anti-persistence; and aging and anti-aging. Thus, the following conclusion is attained.

▶ **Table 5–which was presented with regard to PBM–holds ‘as is’ also with regard to PLM.**

In the transition from PBM to PLM, the following notation adjustments should be made in row #2 of Table 5: elevate the SSBM benchmark to the space-scaled SLS motion; and elevate the TSBM benchmark to the time-scaled SLS motion. (Both these ‘benchmark motions’ were defined in Section 5.3).

With regard to PBM, Section 4.3 presented a statistical approach that leads to Table 5. According to the ‘hotel metaphor’ (which was described at the opening of Section 4): the statistical approach of Section 4.3 is akin to a set of keys that open the doors on the Brown floor alone.

The Brown keys do not open the doors on the Levy floor. Elevating from the Brown floor to the Levy floor, the Brown set of keys should be replaced by a parallel Levy set of keys, as follows.

Replace the PBM variances that appear on the left-hand side of Equation (8) by the corresponding PLM extended variances. This replacement yields the very same right-hand side of Equation (8): $r^{\delta }$.

Replace the PBM variance and conditional variance that appear on the left-hand sides of Equations (9) and (10) by the corresponding PLM extended variance and conditional extended variance (detailed in Table 6). This replacement yields the very same right-hand sides of Equations (9) and (10): $r^{\gamma }$ and $1-r^{\gamma }$, respectively.

Replace the PBM conditional mean that appears on the left-hand side of Equation (11) by the corresponding PLM conditional median (detailed in Table 6). This replacement yields the linear slope $\left(r^{\delta /\lambda }-r^{\gamma /\lambda }\right)/r^{\delta /\lambda }$. In terms of the quantity $u=r^{\delta /\lambda }$–a PLM scale ratio that is the Levy parallel of the PBM standard-deviation ratio $u=r^{\delta /2}$ of Equation (11)–the linear slope admits the very same formulation as deduced from Equation (11): $(u-u^{\gamma /\delta })/u$.

As explained in Section 4.3, the right-hand sides of Equations (8)–(11) lead to Table 5. The replacements noted above yield the very same conclusions as the right-hand sides of Equations (8)–(11). Thus, these replacements describe the transition from the Brown set of keys to the parallel Levy set of keys.

## 6. Recap and Outlook

Introduced recently, power Brownian motion (PBM) and power Levy motion (PLM) are central fractal diffusion models in the Brown and Levy kingdoms, respectively. Indeed, PBM is the unique fractal diffusion model that is Gaussian and Markovian, and PLM is the ‘Levy counterpart’ of PBM.

The PBM and PLM models have a significant potential due to the following combination of facts. On the one hand, these models are analytically simple: it is easy to construct PBM and PLM, and it is easy to track their Markovian evolutions. On the other hand, these models are statistically rich. Indeed, PBM and PLM display an assortment of anomalous behaviors including: sub-diffusion and super-diffusion; aging and anti-aging; and persistence and anti-persistence.

This paper established and presented a socioeconomic-inequality perspective via which: main statistical and temporal behaviors of PBM and PLM are interpreted and scored. This perspective is summarized in Table 1 and Table 5, and the steps leading to this perspective are summarized below.

First, three power-law clocks of PBM and PLM were identified: a diffusion clock, with a positive exponent δ; an inherent clock, with a positive exponent γ; and a relative clock, with the positive exponent γ/δ. In turn, these clocks induce, respectively, three clock ratios.

Second, the axes underpinning the three clock ratios were mapped to the axes underpinning Lorenz curves–which are objects that quantify the distribution of wealth (in human societies) in a universally-calibrated way. In turn, the three clock ratios got mapped to power-law Lorenz curves–which are objects that characterize fractal wealth distributions.

Third, conclusions that follow from the mapping were obtained. Here is a terse summary of main conclusions.

▶ PBM and PLM anomalous behaviors were classified according to three socioeconomic regimes: rich fractality; poor fractality; and perfect equality–which is the socioeconomic benchmark.
▶ The PBM and PLM benchmarks–which correspond to perfect equality–were shown to be: regular diffusion; space-scaled Brown and Levy motions; and time-scaled Brown and Levy motions.
▶ The deviations of PBM and PLM from their benchmarks were quantified by inequality indices–measures that score socioeconomic inequality on a zero-to-one scale.

The inequality indices provide numerical answers to the question: what is the degree-of-anomaly of each of the PBM and PLM anomalous behaviors?

Described in an ‘illustrative nutshell’: the above mapping leads to a vantage point which offers a socioeconomic view of the PBM and PLM landscape. In turn, the view offers a socioeconomic interpretation and scoring of the PBM and PLM anomalous behaviors.

Apparently, this work is the first to apply a socioeconomic-inequality perspective to gauge anomalous behaviors of fractal diffusion models. Possible directions for future research are the following.

As PBM and PLM were introduced only recently, applications of these models are yet to be proposed. One direction for future research is to seek for PBM and PLM applications in the physical sciences, as well as in other fields–e.g.: biology, finance, and engineering. The implementation of the quantitative tools established in this work will be facilitated by such applications.

Another direction for future research is to carry on with the socioeconomic-inequality perspective (from PBM and PLM) to other fractal diffusion models, e.g.: general fractal Brown and Levy motions; fractal random motions that are generated by stochastic differential equations driven by Brown and Levy motions [191]; fractal diffusion models with, so called, diffusive diffusivity [192,193,194]; and multifractal diffusion models [195,196,197].

The latter direction will reveal the scope of the socioeconomic-inequality perspective. Is the scope narrow (applying to PBM and PLM alone)? Or is the scope broad (applying to many more fractal diffusion models)? Perhaps future research will manage to draw a precise chart of the scope’s boundaries.

## Figures and Tables

**Table 1:** Socioeconomic perspective of PBM and PLM. The fractal wealth distributions are parameterized by a positive power *p*, and are classified by the table’s rows as follows: rich fractality when p<1; poor fractality when p>1; and perfect equality when p=1, which is the phase transition between rich fractality and poor fractality. Key parameters of PBM and PLM are the positive exponents δ and γ. The table’s columns set these exponents, as well as their ratio γ/δ, to assume the role of the power *p*. In turn, the table’s cells showcase a unified socioeconomic classification of main statistical and temporal behaviors behaviors of PBM and PLM: sub-diffusion and super-diffusion; aging and anti-aging; sub-linear and super-linear inherent clock; and persistence and anti-persistence. The socioeconomic benchmark is the ‘ground-state’ of perfect equality, and the corresponding PBM and PLM benchmarks are specified in the cells of the perfect-equality row.

	p=δ	p=γ	p=γ/δ
**Rich** **fractality**	$\begin{array}{c} \delta <1 \end{array}$ Sub diff. & Aging	$\begin{array}{c} \gamma <1 \end{array}$ Sub-linear clock	$\begin{array}{c} \gamma <\delta \end{array}$ Persistence
**Perfect** **equality**	$\begin{array}{c} \delta =1 \end{array}$ Regular diff.	$\begin{array}{c} \gamma =1 \end{array}$ Space-scaled motion	$\begin{array}{c} \gamma =\delta \end{array}$ Time-scaled motion
**Poor** **fractality**	$\begin{array}{c} \delta >1 \end{array}$ Super diff. & Anti aging	$\begin{array}{c} \gamma >1 \end{array}$ Super-linear clock	$\begin{array}{c} \gamma >\delta \end{array}$ Anti-persistence

**Table 2:** A ‘map’ of the anomalous behaviors of PBM. The table’s rows address the diffusion behaviors, which are coupled to the aging behaviors. The table’s columns address the persistence behaviors. Each combination of anomalous-diffusion behaviors is characterized by a corresponding combination of the PBM parameters: the positive diffusion exponent δ of Equation (1); and the positive correlation exponent γ of Equation (2).

	Persistent	Anti−Persistent
**Sub-diffusive** **Aging**	γ<δ<1	δ<min(1,γ)
**Super-diffusive** **Anti-aging**	max(1,γ)<δ	1<δ<γ

**Table 3:** Inequality indices. The table’s rows correspond to five different inequality indices. Per each inequality index: the middle column details a general formula that is based on general Lorenz curves; and the right column details a ‘fractal formula’ for the specific power-law Lorenz curve of Equation (6), whose parameter is the positive power *p*. In the general formulae: $x=L_{BU}^{-1}\left(y\right)$ and $x=L_{TD}^{-1}\left(y\right)$ denote, respectively, the inverse functions of the Lorenz curves $y=L_{BU}\left(x\right)$ and $y=L_{TD}\left(x\right)$.

Inequality Index	General Formula	Fractal Formula
**Gini**	$I_{Gini}=\;\int_{0}^{1}\left[L_{TD}\left(x\right)-L_{BU}\left(x\right)\right]dx$	$I_{Gini}=\frac{\left|p-1\right|}{p+1}$
**Vertical** **diameter**	$I_{VD}=L_{TD}\left(\frac{1}{2}\right)-L_{BU}\left(\frac{1}{2}\right)$	$I_{VD}=\left|1-2^{1-p}\right|$
**Horizontal** **diameter**	$I_{HD}=L_{BU}^{-1}\left(\frac{1}{2}\right)-L_{TD}^{-1}\left(\frac{1}{2}\right)$	$I_{HD}=\left|1-2^{1-\frac{1}{p}}\right|$
**Vertical** **Bonferroni**	$I_{VB}=\int_{0}^{1}\frac{x-L_{BU}\left(x\right)}{x}dx$	$I_{VB}=1-\frac{1}{p}$ (when p>1)
**Horizontal** **Bonferroni**	$I_{HB}=\int_{0}^{1}\frac{y-L_{TD}^{-1}\left(y\right)}{y}dy$	$I_{HB}=1-p$ (when p<1)

**Table 4:** The phase transition of fractal wealth distributions. Consider a human society whose average wealth is one, and whose wealth distribution is quantified by the power-law Lorenz curve of Equation (6). Set *W* to be the wealth of a society member that is sampled at random. Per each of the three socioeconomic regimes (noted after Equation (6)), the table’s columns detail: the values of the power parameter *p*; the range of the random variable *W*; and the distribution function of the random variable *W*. Evidently, the threshold p=1 marks a phase transition. Indeed, when crossing the threshold, the statistical distribution of the random variable *W* changes from Pareto (below the threshold) to inverted Pareto (above the threshold).

	Power Parameter	Wealth Range	Statistical Distribution
**Rich** **fractality**	p<1	p<W<∞	$\mathrm{Pr}\left(W>w\right)={\left(\frac{p}{w}\right)}^{1/(1-p)}$
**Perfect** **equality**	p=1	W=1	$\mathrm{Pr}\left(W=1\right)=1$
**Poor** **fractality**	p>1	0<W<p	$\mathrm{Pr}\left(W\leq w\right)={\left(\frac{w}{p}\right)}^{1/(p-1)}$

**Table 5:** PBM clocks and anomalous behaviors. The table’s columns address the three PBM clocks. Per each clock: rows #1 to #3 address the clock classification and the matching socioeconomic regimes; and the table’s cells specify the corresponding PBM exponents and anomalous behaviors. Also, per each clock: row #2 specifies the PBM benchmark (in boldface); and row #4 specifies the fractal Gini gauge that scores the deviation from the benchmark.

	Diffusion Clock	Inherent Clock	Relative Clock
**(1)** **Sub-linear** **Rich fractality**	$\begin{array}{c} \delta <1 \end{array}$ Sub diff. Aging	γ<1	$\begin{array}{c} \gamma <\delta \end{array}$ Persistent
**(2)** **Linear** **Perfect equality**	$\begin{array}{c} \delta =1 \end{array}$ **Regular diff.**	$\begin{array}{c} \gamma =1 \end{array}$ **SSBM**	$\begin{array}{c} \gamma =\delta \end{array}$ **TSBM**
**(3)** **Super-linear** **Poor fractality**	$\begin{array}{c} \delta >1 \end{array}$ Super diff. Anti aging	γ>1	$\begin{array}{c} \gamma >\delta \end{array}$ Anti persistent
**(4)** **Gini gauge**	$G=\frac{\delta -1}{\delta +1}$	$G=\frac{\gamma -1}{\gamma +1}$	$G=\frac{\gamma -\delta }{\gamma +\delta }$

**Table 6:** Conditional SLS distributions. Consider a present time point *t* and a future time point *T* (where 0<t<T), and set r=t/T. Given the present position $X\left(t\right)$, the conditional statistical distributions of the future position $X\left(T\right)$ and of the increment $X\left(T\right)-X\left(t\right)$ are SLS with Levy exponent λ. The table’s columns specify the parameters of conditional SLS distributions: median and extended variance (which is the $\lambda^{\mathrm{th}}$ power of the scale).

	Median	Extended Variance
**Position**	$r^{(\gamma -\delta )/\lambda }\cdot X\left(t\right)$	$\left(1-r^{\gamma }\right)T^{\delta }$
**Increment**	$-\left[1-r^{(\gamma -\delta )/\lambda }\right]\cdot X\left(t\right)$	$\left(1-r^{\gamma }\right)T^{\delta }$

## Data Availability

No new data were created or analyzed in this study.

## References

[1] Mandelbrot B.B. (1982). The Fractal Geometry of Nature.

[2] Feder J. (2013). Fractals.

[3] Barnsley M.F. (2014). Fractals Everywhere.

[4] Gardiner C.W. (1985). Handbook of Stochastic Methods.

[5] Van Kampen N.G. (1992). Stochastic Processes in Physics and Chemistry.

[6] Cussler E.L. (2009). Diffusion: Mass Transfer in Fluid Systems.

[7] Thambynayagam R.K. (2011). The Diffusion Handbook: Applied Solutions for Engineers.

[8] Lamperti J. (1962). Semi-stable stochastic processes. Trans. Am. Math. Soc..

[9] Van der Pas P.W. (1971). The discovery of the Brownian motion. Sci. Hist. Tijdschr. Geschied. Wet. Geneeskd..

[10] Brown R. (1828). A brief account of microscopical observations made in the months of June, July and August 1827, on the particles contained in the pollen of plants; and on the general existence of active molecules in organic and inorganic bodies. Philos. Mag..

[11] Bachelier L. (1900). Theorie de la speculation. Ann. Lecole Norm. Super..

[12] Bachelier L. (2011). Louis Bachelier’s Theory of Speculation: The Origins of Modern Finance.

[13] Einstein A. (1905). Uber die von der molekularkinetischen theorie der warmegeforderte bewegung von in ruhenden flussigkeitensuspendierten teilchen. Ann. Phys..

[14] Von Smoluchowski M. (1906). Zurkinetischentheorie der brownschen molekularbewegung und der suspensionen. Ann. Phys..

[15] Wiener N. (1923). Differential space. J. Math. Physics.

[16] Borodin A.N., Salminen P. (2015). Handbook of Brownian Motion: Facts and Formulae.

[17] Ito K., Henry P. (2012). Diffusion Processes and Their Sample Paths.

[18] Scher H., Lax M. (1972). Continuous time random walk model of hopping transport: Application to impurity conduction. J. Non-Cryst. Solids.

[19] Scher H., Lax M. (1973). Stochastic transport in a disordered solid. I. Theory. Physical Review B.

[20] Scher H., Lax M. (1973). Stochastic transport in a disordered solid. II. Impurity conduction. Phys. Rev. B.

[21] Shlesinger M.F. (1974). Asymptotic solutions of continuous-time random walks. J. Stat. Phys..

[22] Scher H., Montroll E.W. (1975). Anomalous transit-time dispersion in amorphous solids. Phys. Rev. B.

[23] Klafter J., Blumen A., Shlesinger M.F. (1987). Stochastic pathway to anomalous diffusion. Phys. Rev. A.

[24] Bouchaud J.-P., Georges A. (1990). Anomalous diffusion in disordered media: Statistical mechanisms, models and physical applications. Phys. Rep..

[25] Zanette D.H., Alemany P.A. (1995). Thermodynamics of anomalous diffusion. Phys. Rev. Lett..

[26] Metzler R., Klafter J. (2000). The random walk’s guide to anomalous diffusion: A fractional dynamics approach. Phys. Rep..

[27] Sancho J.M., Lacasta A.M., Lindenberg K., Sokolov I.M., Romero A.H. (2004). Diffusion on a solid surface: Anomalous is normal. Phys. Rev. Lett..

[28] Sokolov I.M., Klafter J. (2005). From diffusion to anomalous diffusion: A century after Einstein’s Brownian motion. Chaos Interdiscip. J. Nonlinear Sci..

[29] Klafter J., Sokolov I.M. (2005). Anomalous diffusion spreads its wings. Phys. World.

[30] Khoury M., Lacasta A.M., Sancho J.M., Lindenberg K. (2011). Weak disorder: Anomalous transport and diffusion are normal yet again. Phys. Rev. Lett..

[31] Eliazar I., Klafter J. (2011). Anomalous is ubiquitous. Ann. Phys..

[32] Metzler R., Jeon J.-H., Cherstvy A.G., Barkai E. (2014). Anomalous diffusion models and their properties: Non-stationarity, non-ergodicity, and ageing at the centenary of single particle tracking. Phys. Chem. Chem. Phys..

[33] Klages R., Radons G., Sokolov I.M. (2008). Anomalous Transport.

[34] Evangelista L.R., Lenzi E.K. (2018). Fractional Diffusion Equations and Anomalous Diffusion.

[35] Evangelista L.R., Lenzi E.K. (2023). An Introduction to Anomalous Diffusion and Relaxation.

[36] Giona M., Cairoli A., Klages R. (2022). In the folds of the central limit theorem: Lévy walks, large deviations and higher-order anomalous diffusion. J. Phys. A Math. Theor..

[37] Vilk O., Aghion E., Avgar T., Beta C., Nagel O., Sabri A., Sarfati R., Schwartz D.K., Weiss M., Krapf D. (2022). Unravelling the origins of anomalous diffusion: From molecules to migrating storks. Phys. Rev. Res..

[38] Sposini V., Krapf D., Marinari E., Sunyer R., Ritort F., Taheri F., Selhuber-Unkel C., Benelli R., Weiss M., Metzler R. (2022). Towards a robust criterion of anomalous diffusion. Commun. Phys..

[39] Firbas N., Garibo-I-Orts Ò., Garcia-March M.Á., Conejero J.A. (2023). Characterization of anomalous diffusion through convolutional transformers. J. Phys. A Math. Theor..

[40] Sevilla F.J., Chacón-Acosta G., Sandev T. (2024). Anomalous diffusion of self-propelled particles. J. Phys. A Math. Theor..

[41] Seckler H., Metzler R. (2024). Change-point detection in anomalous-diffusion trajectories utilising machine-learning-based uncertainty estimates. J. Phys. Photon..

[42] Tomovski Z., Górska K., Pietrzak T., Metzler R., Sandev T. (2024). Anomalous and ultraslow diffusion of a particle driven by power-law-correlated and distributed-order noises. J. Phys. A Math. Theor..

[43] Goswami K., Cherstvy A.G., Godec A., Metzler R. (2024). Anomalous diffusion of active Brownian particles in responsive elastic gels: Nonergodicity, non-Gaussianity, and distributions of trapping times. Phys. Rev. E.

[44] Gentili A., Klages R., Volpe G. (2024). Anomalous Diffusion of Superparamagnetic Walkers with Tailored Statistics. arXiv.

[45] Vilk O., Charter M., Toledo S., Barkai E., Nathan R. (2025). Strong anomalous diffusion for free-ranging birds. PRX Life.

[46] Cieśla M., Dybiec B., Krasowska M., Strzelewicz A. (2025). Effective anomalous diffusion in a conical channel. Chaos Interdiscip. J. Nonlinear Sci..

[47] Armstrong S., Vicol V. (2025). Anomalous diffusion by fractal homogenization. Ann. PDE.

[48] Sandev T., Iomin A., Kurths J., Kocarev L. (2025). Shear-driven anomalous diffusion: Memory effects and stochastic resetting. Phys. Fluids.

[49] Eliazar I. (2023). Power Brownian motion. J. Phys. A Math. Theor..

[50] Eliazar I. (2024). Power Brownian Motion: An Ornstein–Uhlenbeck lookout. J. Phys. A Math. Theor..

[51] Eliazar I. (2024). Taylor’s Law from Gaussian diffusions. J. Phys. A Math. Theor..

[52] Eliazar I. (2025). Power Levy motion. I. Diffusion. Chaos Interdiscip. J. Nonlinear Sci..

[53] Eliazar I. (2025). Power Levy motion. II. Evolution. Chaos Interdiscip. J. Nonlinear Sci..

[54] Eliazar I. (2025). Power Levy motion: Correlations and relaxation. Phys. A Stat. Mech. Appl..

[55] Bauer B., Gerhold S. (2020). Self-similar Gaussian Markov processes. arXiv.

[56] Hao L., Naiman D.Q. (2010). Assessing Inequality.

[57] Cowell F. (2011). Measuring Inequality.

[58] Eliazar I. (2016). Harnessing inequality. Phys. Rep..

[59] Eliazar I. (2018). A tour of inequality. Ann. Phys..

[60] Coulter P.B. (2019). Measuring Inequality: A Methodological Handbook.

[61] Eliazar I., Cohen M.H. (2014). Hierarchical socioeconomic fractality: The rich, the poor, and the middle-class. Phys. A Stat. Mech. Appl..

[62] Eliazar I. (2020). Power Laws: A Statistical Trek.

[63] MacKay D.J.C. (1998). Introduction to Gaussian processes. NATOASI Ser. F Comput. Syst. Sci..

[64] Ibragimov I., Rozanov Y. (2012). Gaussian Random Processes.

[65] Lifshits M. (2012). Lectures on Gaussian Processes.

[66] Bertoin J. (1998). Levy Processes.

[67] Ken-Iti S. (1999). Levy Processes and Infinitely Divisible Distributions.

[68] Barndorff-Nielsen O.E., Mikosch T., Resnick S.I. (2001). Levy Processes: Theory and Applications.

[69] Gillespie D.T. (1991). Markov Processes: An Introduction for Physical Scientists.

[70] Liggett T.M. (2010). Continuous Time Markov Processes: An Introduction.

[71] Dynkin E.B. (1989). Theory of Markov Processes. Ann. Probab..

[72] Tsybakov B., Georganas N. (1998). Self-similar processes in communications networks. IEEE Trans. Inf. Theory.

[73] Sheluhin O., Smolskiy S., Osin A. (2007). Self-Similar Processes in Telecommunications.

[74] Embrechts P., Maejima M. (2009). Selfsimilar Processes.

[75] Eliazar I. (2022). Selfsimilar stochastic differential equations. Europhys. Lett..

[76] Donsker M.D. (1951). An invariance principle for certain probability limit theorems. Mem. Amer. Math. Soc..

[77] Whitt W. (2002). Stochastic-Process Limits: An Introduction to Stochastic-Process Limits and Their Application to Queues.

[78] Eliazar I., Shlesinger M.F. (2013). Fractional motions. Phys. Rep..

[79] Lawler G.F., Limic V. (2010). Random Walk: A Modern Introduction.

[80] Klafter J., Sokolov I.M. (2011). First Steps in Random Walks: From Tools to Applications.

[81] Ibe O.C. (2013). Elements of Random Walk and Diffusion Processes.

[82] Shlesinger M.F. (2021). An Unbounded Experience in Random Walks with Applications.

[83] Schwarz W. (2022). Random Walk and Diffusion Models.

[84] Kolmogorov A.N. (1940). The Wiener spiral and some other interesting curves in Hilbert space. Dokl. Akad. Nauk SSSR.

[85] Yaglom A.M. (1958). Correlation theory of processes with stationary increments of order n. Amer. Math. Soc. Transl. Ser. Am. Math. Soc. Providence RI.

[86] Mandelbrot B.B., Van Ness J.W. (1968). Fractional Brownian motions, fractional noises and applications. SIAM Rev..

[87] Batchelor G.K. (1952). Diffusion in a field of homogeneous turbulence: II. The relative motion of particles. Mathematical Proceedings of the Cambridge Philosophical Society.

[88] Monin A.S., Yaglom A.M. (1975). Statistical Fluid Mechanics, Volume II: Mechanics of Turbulence.

[89] Lim S.C., Muniandy S.V. (2002). Self-similar Gaussian processes for modeling anomalous diffusion. Phys. Rev. E.

[90] Chechkin A., Gonchar V. (2001). Fractional Brownian motion approximation based on fractional integration of a white noise. Chaos Solitons Fractals.

[91] Mercik S., Weron K. (2001). Fractional Brownian Motion as a Model of the Self-Similar Ion Channel Kinetics. Acta Phys. Pol. Ser. B.

[92] Magdziarz M., Weron A., Burnecki K., Klafter J. (2009). Fractional Brownian motion versus the continuous-time random walk: A simple test for subdiffusive dynamics. Phys. Rev. Lett..

[93] Jeon J.-H., Metzler R. (2010). Fractional Brownian motion and motion governed by the fractional Langevin equation in confined geometries. Phys. Rev. E.

[94] Burnecki K., Kepten E., Janczura J., Bronshtein I., Garini Y., Weron A. (2012). Universal algorithm for identification of fractional Brownian motion. A case of telomere subdiffusion. Biophys. J..

[95] Berzin C., Latour A., León J.R. (2014). Inference on the Hurst Parameter and the Variance of Diffusions Driven by Fractional Brownian Motion.

[96] Sikora G., Burnecki K., Wyłomańska A. (2017). Mean-squared-displacement statistical test for fractional Brownian motion. Phys. Rev. E.

[97] Guggenberger T., Pagnini G., Vojta T., Metzler R. (2019). Fractional Brownian motion in a finite interval: Correlations effect depletion or accretion zones of particles near boundaries. New J. Phys..

[98] Guggenberger T., Chechkin A., Metzler R. (2021). Fractional Brownian motion in superharmonic potentials and non-Boltzmann stationary distributions. J. Phys. A Math. Theor..

[99] Cherstvy A.G., Wang W., Metzler R., Sokolov I.M. (2021). Inertia triggers nonergodicity of fractional Brownian motion. Phys. Rev. E.

[100] Wang W., Cherstvy A.G., Kantz H., Metzler R., Sokolov I.M. (2021). Time averaging and emerging nonergodicity upon resetting of fractional Brownian motion and heterogeneous diffusion processes. Phys. Rev. E.

[101] Balcerek M., Burnecki K., Thapa S., Wyłomańska A., Chechkin A. (2022). Fractional Brownian motion with random Hurst exponent: Accelerating diffusion and persistence transitions. Chaos Interdiscip. J. Nonlinear Sci..

[102] Khadem S.M.J., Klages R., Klapp S.H.L. (2022). Stochastic thermodynamics of fractional Brownian motion. Phys. Rev. Res..

[103] Liang Y., Wang W., Metzler R., Cherstvy A.G. (2023). Anomalous diffusion, nonergodicity, non-Gaussianity, and aging of fractional Brownian motion with nonlinear clocks. Phys. Rev. E.

[104] Liang Y., Wang W., Metzler R., Cherstvy A.G. (2023). Nonergodicity of confined superdiffusive fractional Brownian motion. Phys. Rev. E.

[105] Hartmann A.K., Meerson B. (2024). First-passage area distribution and optimal fluctuations of fractional Brownian motion. Phys. Rev. E.

[106] Pacheco-Pozo A., Krapf D. (2024). Fractional Brownian motion with fluctuating diffusivities. Phys. Rev. E.

[107] Liang Y., Wang W., Metzler R. (2024). Aging and confinement in subordinated fractional Brownian motion. Phys. Rev. E.

[108] Wei Q., Wang W., Tang Y., Metzler R., Chechkin A. (2025). Fractional Langevin equation far from equilibrium: Riemann-Liouville fractional Brownian motion, spurious nonergodicity, and aging. Phys. Rev. E.

[109] Woszczek H., Wyłomańska A., Chechkin A. (2025). Riemann–Liouville fractional Brownian motion with random Hurst exponent. Chaos: Interdiscip. J. Nonlinear Sci..

[110] Régnier L., Dolgushev M., Bénichou O. (2025). Visitation Dynamics of d-Dimensional Fractional Brownian Motion. Phys. Rev. Lett..

[111] Jeon J.-H., Chechkin A.V., Metzler R. (2014). Scaled Brownian motion: A paradoxical process with a time dependent diffusivity for the description of anomalous diffusion. Phys. Chem. Chem. Phys..

[112] Thiel F., Sokolov I.M. (2014). Scaled Brownian motion as a mean-field model for continuous-time random walks. Phys. Rev. E.

[113] Safdari H., Chechkin A.V., Jafari G.R., Metzler R. (2015). Aging scaled Brownian motion. Phys. Rev. E.

[114] Safdari H., Cherstvy A.G., Chechkin A.V., Thiel F., Sokolov I.M., Metzler R. (2015). Quantifying the non-ergodicity of scaled Brownian motion. J. Phys. A Math. Theor..

[115] Bodrova A.S., Chechkin A.V., Cherstvy A.G., Metzler R. (2015). Ultraslow scaled Brownian motion. New J. Phys..

[116] Bodrova A.S., Chechkin A.V., Cherstvy A.G., Safdari H., Sokolov I.M., Metzler R. (2016). Underdamped scaled Brownian motion: (Non-)existence of the overdamped limit in anomalous diffusion. Sci. Rep..

[117] Safdari H., Cherstvy A.G., Chechkin A.V., Bodrova A., Metzler R. (2017). Aging underdamped scaled Brownian motion: Ensemble- and time-averaged particle displacements, nonergodicity, and the failure of the overdamping approximation. Phys. Rev. E.

[118] Bodrova A.S., Chechkin A.V., Sokolov I.M. (2019). Scaled Brownian motion with renewal resetting. Phys. Rev. E.

[119] Dos S., Maike A.F., Menon L. (2021). Random diffusivity models for scaled Brownian motion. Chaos Solitons Fractals.

[120] dos Santos M., Menon L., Cius D. (2022). Superstatistical approach of the anomalous exponent for scaled Brownian motion. Chaos Solitons Fractals.

[121] Wang W., Metzler R., Cherstvy A.G. (2022). Anomalous diffusion, aging, and nonergodicity of scaled Brownian motion with fractional Gaussian noise: Overview of related experimental observations and models. Phys. Chem. Chem. Phys..

[122] Li Y., Suleiman K., Xu Y. (2024). Anomalous diffusion, non-Gaussianity, nonergodicity, and confinement in stochastic-scaled Brownian motion with diffusing diffusivity dynamics. Phys. Rev. E.

[123] Suleiman K., Li Y., Xu Y. (2024). Anomalous non-Gaussian diffusion of scaled Brownian motion in a quenched disorder environment. J. Phys. A Math. Theor..

[124] Sánchez-Monroy J., Riascos-Ochoa J., Bustos A. (2024). Parameter estimation in the stochastic SIR model via scaled geometric Brownian motion. Chaos Solitons Fractals.

[125] Sevilla F.J., Valdés-Gómez A., Torres-Carbajal A. (2024). Anomalous diffusion of scaled Brownian tracers. Phys. Rev. E.

[126] Woszczek H., Chechkin A., Wyłomańska A. (2024). Scaled Brownian motion with random anomalous diffusion exponent. Commun. Nonlinear Sci. Numer. Simul..

[127] Shiryaev A.N. (1999). Essentials of Stochastic Finance: Facts, Models, Theory.

[128] Lorenz M.O. (1905). Methods of measuring the concentration of wealth. Publ. Am. Stat. Assoc..

[129] Gastwirth J.L. (1971). A general definition of the Lorenz curve. Econom. J. Econom. Soc..

[130] Chotikapanich D. (2008). Modeling Income Distributions and Lorenz Curves.

[131] Arnold B.C., Sarabia J.M. (2018). Majorization and the Lorenz Order with Applications in Applied Mathematics and Economics.

[132] Eliazar I. (2015). The sociogeometry of inequality: Part I. Phys. A Stat. Mech. Its Appl..

[133] Eliazar I. (2015). The sociogeometry of inequality: Part II. Phys. A: Stat. Mech. Its Appl..

[134] Gini C. (1914). Sulla misura della concentrazione e della variabilita dei caratteri. Atti R. Ist. Veneto Sci. Ed Arti.

[135] Gini C. (1921). Measurement of inequality of incomes. Econ. J..

[136] Yitzhaki S., Schechtman E. (2012). The Gini Methodology: A Primer on a Statistical Methodology.

[137] Giorgi G.M., Gigliarano C. (2017). The Gini concentration index: A review of the inference literature. J. Econ. Surv..

[138] Giorgi G.M., Atkinson P., Delamont S., Cernat A., Sakshaug J.W., Williams R.A. (2020). Gini Coefficient. SAGE Research Methods Foundations.

[139] Yitzhaki S. (1998). More than a dozen alternative ways of spelling Gini. Res. Econ. Inequal..

[140] Eliazar I. (2024). Beautiful Gini. Metron.

[141] Eliazar I., Giorgi G.M. (2020). From Gini to Bonferroni to Tsallis: An inequality-indices trek. Metron.

[142] Bonferroni C.E. (1930). Elementi di Statistica Generale.

[143] Nygard F., Sandstrum A. (1981). Measuring Income Inequality.

[144] Dagum C., Zenga M. (2012). Income and Wealth Distribution, Inequality and Poverty: Proceedings of the Second International Conference on Income Distribution by Size: Generation, Distribution, Measuremente and Applications, Held at the University of Pavia, Italy, 28–30 September 1989.

[145] Giorgi G.M., Crescenzi M. (2001). A proposal of poverty measures based on the Bonferroni inequality index. Metron.

[146] Giorgi G.M., Nadarajah S. (2010). Bonferroni and Gini indices for various parametric families of distributions. Metron.

[147] Imedio-Olmedo L.J., Barcena-Martin E., Parrado-Gallardo E.M. (2011). A class of Bonferroni inequality indices. J. Public Econ. Theory.

[148] Imedio-Olmedo L.J., Parrado-Gallardo E.M., Barcena-Martin E. (2012). Income inequality indices interpreted as measures of relative deprivation/satisfaction. Soc. Indic. Res..

[149] Barcena-Martin E., Silber J. (2013). On the generalization and decomposition of the Bonferroni index. Soc. Choice Welfare.

[150] Arnold B.C. (2015). Pareto Distributions.

[151] Pietra G. (1914). Atti del Reale Istituto Veneto di Scienze, Lettere ed Arti, Tomo LXXIV, Parte II, Tomo LXXIV.

[152] Zolotarev V.M. (1986). One-Dimensional Stable Distributions.

[153] Borak S., Hardle W., Weron R. (2005). Stable Distributions.

[154] Nolan J.P. (2020). Univariate Stable Distributions.

[155] Eliazar I. (2025). Brown and Levy Steady-State Motions. Entropy.

[156] Shlesinger M.F., Klafter J. (1986). Levy walks versus Levy flights. On Growth and Form: Fractal and Non-Fractal Patterns in Physics.

[157] Shlesinger M.F., Klafter J., West B.J. (1986). Levy walks with applications to turbulence and chaos. Phys. A Stat. Mech. Appl..

[158] Allegrini P., Grigolini P., West B.J. (1996). Dynamical approach to Lévy processes. Phys. Rev. E.

[159] Shlesinger M.F., West B.J., Klafter J. (1987). Lévy dynamics of enhanced diffusion: Application to turbulence. Phys. Rev. Lett..

[160] Uchaikin V.V. (2003). Self-similar anomalous diffusion and Levy-stable laws. Physics-Uspekhi.

[161] Chechkin A.V., Gonchar V.Y., Klafter J., Metzler R. (2006). Fundamentals of Levy flight processes. Fractals, Diffusion, and Relaxation in Disordered Complex Systems: Advances in Chemical Physics Part B.

[162] Metzler R., Chechkin A.V., Gonchar V.Y., Klafter J. (2007). Some fundamental aspects of Lévy flights. Chaos Solitons Fractals.

[163] Chechkin A.V., Metzler R., Klafter J., Gonchar V.Y. (2008). Introduction to the theory of Levy flights. Anomalous Transport: Foundations and Applications.

[164] Dubkov A.A., Spagnolo B., Uchaikin V.V. (2008). Levy flight superdiffusion: An introduction. Int. Bifurc. Chaos.

[165] Schinckus C. (2013). How physicists made stable Levy processes physically plausible. Braz. J. Phys..

[166] Zaburdaev V., Denisov S., Klafter J. (2015). Levy walks. Rev. Mod. Phys..

[167] RReynolds A.M. (2018). Current status and future directions of Lévy walk research. Biol. Open.

[168] Abe M.S. (2020). Functional advantages of Lévy walks emerging near a critical point. Proc. Natl. Acad. Sci. USA.

[169] Xu P., Zhou T., Metzler R., Deng W. (2020). Lévy walk dynamics in an external harmonic potential. Phys. Rev. E.

[170] Levernier N., Textor J., Bénichou O., Voituriez R. (2020). Inverse square Levy walks are not optimal search strategies for *d*≥2. Phys. Rev. Lett..

[171] Garg K., Kello C.T. (2021). Efficient Lévy walks in virtual human foraging. Sci. Rep..

[172] Mukherjee S., Singh R.K., James M., Ray S.S. (2021). Anomalous diffusion and Levy walks distinguish active from inertial turbulence. Phys. Rev. Lett..

[173] Majumdar S.N., Mounaix P., Sabhapandit S., Schehr G. (2021). Record statistics for random walks and Lévy flights with resetting. J. Phys. A Math. Theor..

[174] Gunji Y.-P., Kawai T., Murakami H., Tomaru T., Minoura M., Shinohara S. (2021). Levy walk in swarm models based on Bayesian and inverse Bayesian inference. Comput. Struct. Biotechnol. J..

[175] Park S., Thapa S., Kim Y.-J., A Lomholt M., Jeon J.-H. (2021). Bayesian inference of Lévy walks via hidden Markov models. J. Phys. A Math. Theor..

[176] Guinard B., Korman A. (2021). Intermittent inverse-square Lévy walks are optimal for finding targets of all sizes. Sci. Adv..

[177] Clementi A., D’AMore F., Giakkoupis G., Natale E. Search via parallel Levy walks on Z2. Proceedings of the 2021 ACM Symposium on Principles of Distributed Computing.

[178] Aghion E., Meyer P.G., Adlakha V., Kantz H., E Bassler K. (2021). Moses, Noah and Joseph effects in Lévy walks. New J. Phys..

[179] Cleland J.D., Williams M.A.K. (2022). Analytical Investigations into Anomalous Diffusion Driven by Stress Redistribution Events: Consequences of Lévy Flights. Mathematics.

[180] Padash A., Sandev T., Kantz H., Metzler R., Chechkin A.V. (2022). Asymmetric Levy flights are more efficient in random search. Fractal Fract..

[181] Romero-Ruiz A., Rivero M.J., Milne A., Morgan S., Filho P.M., Pulley S., Segura C., Harris P., Lee M.R., Coleman K. (2023). Grazing livestock move by Lévy walks: Implications for soil health and environment. J. Environ. Manag..

[182] De Bruyne B., Majumdar S.N., Schehr G. (2023). Universal order statistics for random walks & Levy flights. J. Stat. Phys..

[183] Kukushkin A.B., Kulichenko A.A. (2023). Levy Walks as a Universal Mechanism of Turbulence Nonlocality. Foundations.

[184] Sakiyama T., Okawara M. (2023). A short memory can induce an optimal Levy walk. World Conference on Information Systems and Technologies.

[185] Zbik B., Dybiec B. (2024). Levy flights and Levy walks under stochastic resetting. Phys. Rev. E.

[186] Radice M., Cristadoro G. (2024). Optimizing leapover lengths of Levy flights with resetting. Phys. Rev. E.

[187] Gautam D., Meena H., Matheshwaran S., Chandran S. (2024). Harnessing density to control the duration of intermittent Lévy walks in bacterial turbulence. Phys. Rev. E.

[188] Kincses D., Nagy M., Csanád M. (2025). Lévy walk of pions in heavy-ion collisions. Commun. Phys..

[189] Aerdker S., Merten L., Effenberger F., Fichtner H., Tjus J.B. (2024). Superdiffusion of energetic particles at shocks: A Lévy flight model for acceleration. Astron. Astrophys..

[190] Teuerle M.A. (2025). Taking a break: The impact of rests on Lévy walks. Chaos Interdiscip. J. Nonlinear Sci..

[191] Eliazar I., Arutkin M. (2025). Designing selfsimilar diffusions. Phys. A Stat. Mech. Its Appl..

[192] Jain R., Sebastian K.L. (2017). Diffusing diffusivity: A new derivation and comparison with simulations. J. Chem..

[193] Chechkin A.V., Seno F., Metzler R., Sokolov I.M. (2017). Brownian yet non-Gaussian diffusion: From superstatistics to subordination of diffusing diffusivities. Phys. Rev. X.

[194] Wang W., Wei Q., Chechkin A.V., Metzler R. (2025). Different behaviors of diffusing diffusivity dynamics based on three different definitions of fractional Brownian motion. Phys. Rev. E.

[195] Saichev A.I., Filimonov V.A. (2007). On the spectrum of multifractal diffusion process. J. Exp. Theor..

[196] Sandev T., Chechkin A.V., Korabel N., Kantz H., Sokolov I.M., Metzler R. (2015). Distributed-order diffusion equations and multifractality: Models and solutions. Phys. Rev. E.

[197] Balcerek M., Thapa S., Burnecki K., Kantz H., Metzler R., omańska A.W., Chechkin A. (2025). Multifractional Brownian motion with telegraphic, stochastically varying exponent. Phys. Rev. Lett..

